# Weekly trends in emergency department visits, hospitalizations, and mortality in patients with end-stage kidney disease: nationwide registry analysis

**DOI:** 10.1371/journal.pone.0339905

**Published:** 2026-02-20

**Authors:** Hye Eun Yoon, AJin Cho, Seon A. Jeong, Wookjin Choi, Dai Hai Choi, Hayne Cho Park, Jung Eon Kim, Young-Ki Lee, Kyung Don Yoo, on behalf of the Korean Society of Nephrology Disaster Preparedness and Response Committee

**Affiliations:** 1 Department of Internal Medicine, Seoul St Mary’s Hospital, College of Medicine, The Catholic University of Korea, Seoul, Republic of Korea; 2 Transplantation Research Center, College of Medicine, The Catholic University of Korea, Seoul, Republic of Korea; 3 Department of Internal Medicine, Konkuk University Medical Center, Konkuk University School of Medicine, Seoul, Republic of Korea; 4 Korean Society of Nephrology, Seoul, Republic of Korea; 5 Department of Emergency Medicine, Ulsan University Hospital, University of Ulsan College of Medicine, Ulsan, Republic of Korea; 6 Department of Emergency Medicine, CHA University Graduate School of Medicine, Pocheon, South Korea; 7 Department of Internal Medicine, Kangnam Sacred Heart Hospital, Hallym University College of Medicine, Seoul, Republic of Korea; 8 Hallym Kidney Research Institute, Hallym University College of Medicine, Seoul, Republic of Korea; 9 National Emergency Medical Center, National Medical Center, Seoul, Republic of Korea; 10 Department of Internal Medicine, Ulsan University Hospital, University of Ulsan College of Medicine, Ulsan, Republic of Korea; 11 Basic-Clinical Convergence Research Institute, University of Ulsan, Ulsan, Republic of Korea; KPC Medical College and Hospital, INDIA

## Abstract

Patients with end-stage kidney disease (ESKD) undergoing thrice-weekly hemodialysis may experience higher mortality rates following their longest dialysis-free interval. However, information on weekly patterns of emergency department (ED) visits and outcomes in patients with ESKD compared with those without chronic kidney disease (CKD) is limited. We aimed to examine ED visit patterns among patients with ESKD throughout the week in the Republic of Korea and assess whether weekend admissions correlate with higher ED utilization and mortality rates. To this end, we retrospectively analyzed data from the National Emergency Department Information System in the Republic of Korea from January 2018 to December 2021. ED visits, hospitalizations, and in-hospital mortality were assessed daily. Logistic regression analyses were used to adjust for demographic and clinical factors, including the Korean Triage and Acuity Scale. Overall, 159,456 ED visits by patients with ESKD and 21,547,014 ED visits by those without CKD were included. Compared with the non-CKD group, patients with ESKD were older, mostly men, and presented with more severe conditions at ED admission. A distinct weekly pattern emerged among patients with ESKD, with the highest ED visit rates on Mondays (19.9%) and Tuesdays (15.8%), whereas those with non-CKD most frequently visited on Sundays (19.1%) and Saturdays (16.2%). Hospitalization rates were substantially higher among patients with ESKD (66.5%) than among those with non-CKD (21.3%), peaking on Wednesdays (67.2%) and Thursdays (67.4%). Moreover, patients with ESKD had a higher in-hospital mortality rate (9.2%) than those without CKD (5.1%), particularly on Wednesdays (9.7%) and Thursdays (9.4%). Our findings indicate that patients with ESKD exhibit pronounced weekly patterns in ED visits, hospitalizations, and mortality compared with those without CKD. Notably, ED visits among patients with ESKD were highest on Mondays and Tuesdays, whereas hospitalization and mortality rates peaked on Wednesdays and Thursdays.

## Introduction

Emergency department (ED) utilization is notably high among patients with end-stage kidney disease (ESKD) owing to their chronic condition and frequent dialysis-related complications. Patients on hemodialysis are particularly vulnerable, with ED visitation rates significantly exceeding those in general population [1,2]. Patients receiving maintenance hemodialysis are at high risk of acute care utilization even after hospital discharge, with nearly one in three experiencing rehospitalization or an ED visit within 30 days [3]. Han et al. [1] reported that patients undergoing dialysis visited the ED 8.5 times more frequently, often for non-kidney-related issues, reflecting their high comorbidities burden. Weekend admissions present unique challenges for patients undergoing dialysis. Sakhuja et al. [4] reported increased mortality in patients on hemodialysis admitted over weekends; similarly, based on claim data from the United States, the authors found that patients undergoing maintenance dialysis admitted over weekends had higher mortality rates and longer hospital stays than those admitted on weekdays. Zhang et al. [5] further identified a “post-weekend effect” where ED visits and hospitalizations spike after weekends or extended interdialytic intervals, aligning with the “sawtooth” pattern of ED utilization on Mondays.

These trends are exacerbated by limited weekend access to routine care and healthcare disparities. For instance, studies highlight high ED visit rates among patients undergoing dialysis who miss treatments or experience prolonged interdialytic intervals owing to logistic and healthcare access barriers [6,7]. Social determinants of health, including socioeconomic status and racial disparities, further impact ED utilization, because patients undergoing dialysis from lower-income or minority communities often lack access to regular healthcare support [8,9].

In this study, we aimed to examine ED visit patterns among patients with ESKD across different days of the week in the Republic of Korea and assess whether weekend admissions correlate with higher ED utilization and mortality rates. Accordingly, we used 2018–2021 data from the National Emergency Department Information System (NEDIS). Our findings contribute to ongoing discussions on the necessity of healthcare system improvements to mitigate weekend disparities and optimize outcomes in patients with ESKD. This research expands upon existing studies by integrating insights specific to the Korean healthcare context and seeks to inform policy and resource allocation to better support patients with ESKD.

## Materials and methods

### Study design and data sources

In this retrospective, nationwide cohort study, we used the NEDIS database, a nationwide database operated by the National Emergency Medical Center in Korea, to obtain information on ED visits at regional and local emergency medical centers from January 1, 2018, to December 31, 2021. In Korea, the Emergency Medical Act collects real-time information on medical treatment transmitted by emergency medical institutions nationwide. The NEDIS is operated to lay the foundation for building an advanced emergency medical system and provide a basis for research and policy formulation on emergency medical care. Since 2016, over 95% of the nation’s emergency medical centers have participated in the database, with approximately 400 hospitals transmitting patient information from ED visits to the NEDIS. The NEDIS contains comprehensive information on ED visits across the Republic of Korea, including patient demographics, triage level, diagnosis, treatment, and outcomes. The demographic characteristics (age and sex), insurance status, and clinical information about ED visits have been detailed in previous studies [10–12].

### Ethics statement

This study adhered to the principles of the Declaration of Helsinki. The database was fully anonymized, and the Ethics Committee of Ulsan University Hospital Institutional Review Board waived the requirement for informed consent (IRB Nos. UUH2024-01-017 and UUH2023-10-034). This was a retrospective study utilizing publicly available data and did not involve direct interaction with human subjects [13].

### Study population

**Fig 1:**
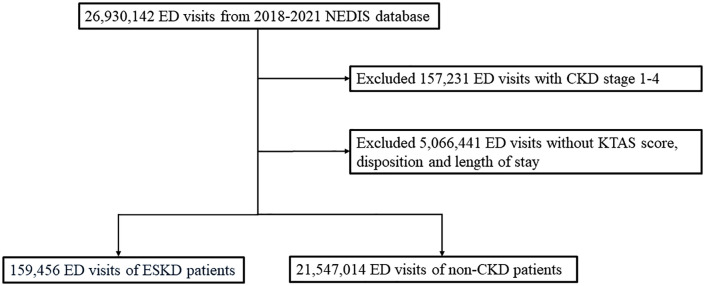
Flowchart of the study population. ED, Emergency Department; NEDIS, National Emergency Department Information System; CKD, Chronic Kidney Disease; KTAS, Korean Triage and Acuity Scale; ESKD, End-Stage Kidney Disease.

We included all ED visitors aged >20 years. The initial NEDIS dataset (2018–2021) comprised 26,930,142 ED visits. ESKD was defined as stage 5 chronic kidney disease (CKD), regardless of whether the patient was on hemodialysis or peritoneal dialysis. ED visits for ESKD were identified using a primary or secondary ED diagnosis (code N18.5 on the International Classification of Diseases, Tenth Revision, Clinical Modification [ICD-10-CM]). To focus on the ED utilization among ESKD patients, 157,231 ED visits of patients with CKD stages 1–4 were excluded, because morbidity and mortality in patients with ESKD are different from those in patients with CKD stages 1–4. We also excluded 5,066,441 visits with no information on the Korean Triage and Acuity Scale (KTAS), ED outcomes, or length of stay. Finally, we included 159,456 ED visits of patients with ESKD and 21,547,014 of those with normal kidney function (non-CKD group) (Fig 1).

### Variables and study outcomes

Demographic characteristics (age and sex), insurance status, and clinical information on ED visits were collected. ED utilization included hospital service level, mode of arrival (including direct visit, transferred from another hospital, and transferred from outpatient), length of stay, transport type (119 ambulances, other ambulance, non-ambulance, and unknown), Korean Triage and Acuity Scale (KTAS), and outcome after the ED visit. The KTAS categorizes patients presenting to the ED according to symptoms, urgency and priority; it was developed through the Korean Emergency Patient Severity Classification System Standardization Study from 2012 to 2015 [14] and consists of a five-level system: 1 (resuscitation), 2 (emergency), 3 (urgent), 4 (less urgent), and 5 (non-emergent). These levels are determined by symptoms, vital signs, and primary call. The Ministry of Health and Welfare designates hospital service levels based on function and size. Information about clinical outcomes, such as discharges and hospitalizations after an ED visit, was included. Based on the leading diagnoses of ED visits, we identified the causes of ED visits, including heart failure and pulmonary edema, ischemic heart disease, cerebrovascular disease, infection, electrolyte imbalance, vascular access, gastrointestinal problems, fracture, and others (S1 Table). Study outcomes included hospitalizations and in-hospital mortality after ED visits for each weekday. NEDIS data provides information about the patient’s status after an ED visit—whether they are discharged home or admitted to the hospital. If the patient is admitted to a general ward or intensive care unit, the database provides clinical outcomes such as discharge or death.

### Statistical analysis

Patients were categorized by weekly ED visit patterns (Monday-Tuesday, Wednesday-Thursday, and Friday-Sunday) or specific visit days. Categorical variables were compared between groups using chi-square tests. Continuous variables were compared between groups using t-tests or analysis of variance.

Data are presented as frequencies (percentages) and means (standard deviations). Logistic regression analysis adjusted for age, sex, insurance status, hospital service level, and KTAS classification was used to analyze the risk of hospitalization and mortality in patients with ESKD, expressed as odds ratios (ORs) with 95% confidence intervals (CIs). All tests were two-tailed, with statistical significance set at p < 0.05. Analyses were conducted using R version 4.0.5 (R Foundation for Statistical Computing, Vienna, Austria; https://www.R-project.org/).

## Results

### Baseline characteristics of the weekly pattern groups

Fig 1 outlines the selection process for the study population from the NEDIS database between 2018 and 2021. Among the 26,772,911 ED visits initially identified, 172,668 visits of patients with CKD stages 1–4 and 5,066,441 with incomplete data, including KTAS scores, disposition, or length of stay, were excluded. The final dataset comprised 159,456 ED visits of patients with ESKD and 21,547,014 ED visits of patients without CKD (Fig 1). Table 1 details the baseline characteristics of ED visits of patients with ESKD, categorized into three weekly pattern groups (Monday-Tuesday, Wednesday-Thursday, and Friday-Sunday). Significant demographic and temporal variations were observed. Younger adults aged 20–29 years constituted 1.14% of the total visits (n = 1,818), with a slightly higher proportion on Friday-Sunday (1.21%) than on Monday-Tuesday (1.06%) and Wednesday-Thursday (1.16%). The 30–39 years old age group accounted for 3.35% of all visits (n = 5,343), with the highest proportion on Friday-Sunday (3.51%), followed by Monday-Tuesday (3.11%) and Wednesday-Thursday (3.44%). Older age groups (40–49, 50–59, 60–69, 70–79, and >80 years old) showed similar distribution patterns, reflecting subtle variations in the timing of ED visits. The highest proportion of ED visits was observed on Monday-Tuesday (35.7%; n = 56,907), followed by that on Friday-Sunday (36.8%; n = 58,721). Wednesday-Thursday accounted for the lowest percentage, with 27.5% (n = 43,828) of visits.

**Table 1:** Baseline characteristics of the weekly pattern groups for ESKD emergency department visits.

Variables		Total	Mon-Tue	Wed-Thu	Fri-Sun	p-value	SMD
Number of emergency department visits		159,456	56,907	43,828	58,721		
Age (years)						<0.001	0.052
20–29		1,818 (1.14)	601 (1.06)	507 (1.16)	710 (1.21)		
30–39		5,343 (3.35)	1,771 (3.11)	1,508 (3.44)	2,064 (3.51)		
40–49		13,041 (8.18)	4,339 (7.62)	3,657 (8.34)	5,045 (8.59)		
50–59		28,992 (18.18)	9,903 (17.40)	7,893 (18.01)	11,196 (19.07)		
60–69		41,024 (25.73)	14,892 (26.17)	11,044 (25.20)	15,088 (25.69)		
70–79		42,165 (26.44)	15,544 (27.31)	11,699 (26.69)	14,922 (25.41)		
≥ 80		27,073 (16.98)	9,857 (17.32)	7,520 (17.16)	9,696 (16.51)		
Sex						0.105	0.009
Men		91,576 (57.43)	24,407 (42.89)	18,509 (42.23)	24,964 (42.51)		
Women		67,880 (42.57)	32,500 (57.11)	25,319 (57.77)	33,757 (57.49)		
Insurance status						0.008	0.019
National health insurance		120,661 (75.67)	13,149 (23.11)	10,164 (23.19)	13,820 (23.54)		
Medical aid		37,133 (23.29)	43,225 (75.96)	33,183 (75.71)	44,253 (75.36)		
Others		988 (0.62)	316 (0.56)	306 (0.70)	366 (0.62)		
Uninsured		367 (0.23)	119 (0.21)	100 (0.23)	148 (0.25)		
Unknown		307 (0.19)	98 (0.17)	75 (0.17)	134 (0.23)		
Hospital service level		62,728 (39.34)				<0.001	0.022
I (REMC)		62,728 (39.34)	22,443 (39.44)	17,270 (39.40)	23,015 (39.19)		
II (CEMC)		84,281 (52.86)	30,166 (53.01)	23,292 (53.14)	30,823 (52.49)		
III (CEMI)		12,447 (7.81)	4,298 (7.55)	3,266 (7.45)	4,883 (8.32)		
Route of arrival						<0.001	0.163
Direct visit		105,588 (66.22)	36,123 (63.48)	27,364 (62.43)	42,101 (71.70)		
Transferred from other hospital		40,377 (25.32)	14,912 (26.20)	11,821 (26.97)	13,644 (23.24)		
Transferred from outpatient clinic		13,366 (8.38)	5,828 (10.24)	4,608 (10.51)	2,930 (4.99)		
Other		123 (0.08)	43 (0.08)	35 (0.08)	45 (0.08)		
Unknown		2 (0.00)	1 (0.00)	0 (0.00)	1 (0.00)		
KTAS classification						<0.001	0.070
Resuscitation		5,928 (3.72)	2,451 (4.31)	1,377 (3.14)	2,100 (3.58)		
Emergent		23,696 (14.86)	8,994 (15.80)	6,092 (13.90)	8,610 (14.66)		
Urgent		89,205 (55.94)	31,835 (55.94)	24,713 (56.39)	32,657 (55.61)		
Less urgent		28,790 (18.06)	9,614 (16.89)	8,083 (18.44)	11,093 (18.89)		
Non urgent		11,837 (7.42)	4,013 (7.05)	3,563 (8.13)	4,261 (7.26)		
Length of stay (h)	Mean (SD)	8.11 (10.23)	8.72 (10.37)	8.32 (10.28)	7.35 (10.00)	<0.001	0.090
Disposition						<0.001	0.049
Discharge home		49,519 (31.05)	17,229 (30.28)	13,336 (30.43)	18,954 (32.28)		
Transfer to other hospital		2,871 (1.80)	1,068 (1.88)	777 (1.77)	1,026 (1.75)		
Admission							
General ward		79,325 (49.75)	28,543 (50.16)	22,482 (51.30)	28,300 (48.19)		
ICU		26,778 (16.79)	9,694 (17.03)	7,006 (15.99)	10,078 (17.16)		
Other		17 (0.01)	7 (0.01)	6 (0.01)	4 (0.01)		
Died in ED		688 (0.43)	269 (0.47)	162 (0.37)	257 (0.44)		
Other/unknown		258 (0.16)	97 (0.17)	59 (0.13)	102 (0.17)		
Transportation						<0.001	0.058
119 ambulances		41,467 (26.01)	15,223 (26.75)	10,514 (23.99)	15,730 (26.79)		
Other ambulance		4,191 (2.63)	6,735 (11.84)	5,353 (12.21)	6,316 (10.76)		
Non-ambulance		18,404 (11.54)	1,532 (2.69)	1,197 (2.73)	1,462 (2.49)		
Unknown		95,394 (59.82)	33,417 (58.72)	26,764 (61.07)	35,213 (59.97)		

CKD, chronic kidney disease; REMC, Regional Emergency Medical Center; CEMC, Community Emergency Medical Center; CEMI, Community Emergency Medical Institute; ICU, intensive care unit; ED, emergency department; SD, standard deviation; KTAS, Korean Triage and Acuity Scale; ESKD, end-stage kidney disease.

### Weekly distribution of emergency department visits, hospitalizations, and mortality in end-stage kidney disease patients

**Fig 2:**
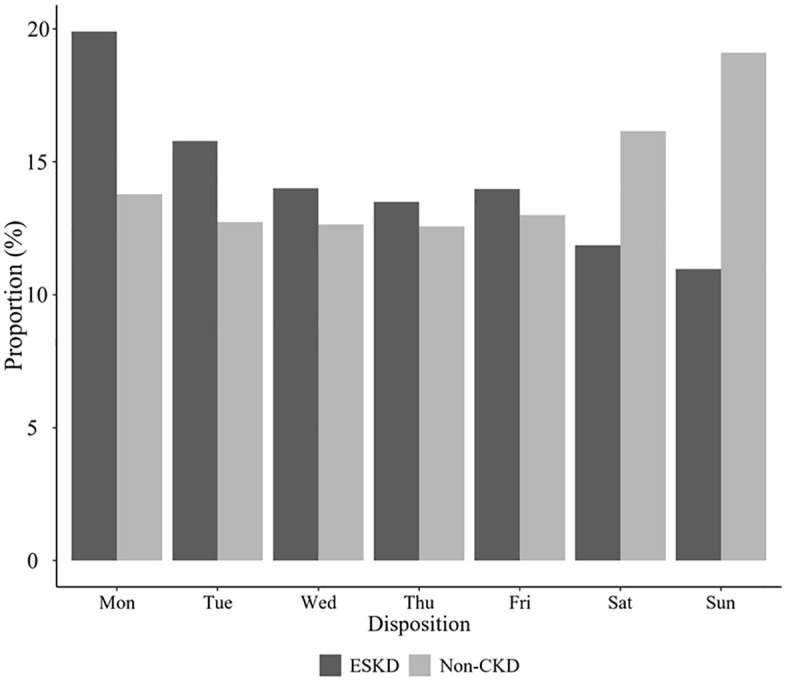
Weekly distribution of emergency department visits of patients with end-stage kidney disease and those with normal kidney function.

Fig 2 illustrates the weekly distribution of ED visits among patients with ESKD and without CKD (Fig 2). ED visits of patients with ESKD followed a distinct weekly pattern, with the highest proportion observed on Mondays at 19.9% (n = 31,735), followed by Tuesdays at 15.79% (n = 25,172). These proportions declined steadily through Wednesday (14.0%, n = 22,325), Thursday (13.49%, n = 21,503), and Friday (13.98%, n = 22,298). Weekend visits were markedly lower, with Saturdays at 11.87% (n = 18,931) and Sundays the lowest at 10.97% (n = 17,492). Conversely, patients without CKD exhibited a more balanced weekly distribution, with the highest proportion of visits on Sunday (19.11%, n = 4,118,366) and Saturday (16.16%, n = 3,481,466); additionally, weekday visits in this group ranged from 13.78% (n = 2,969,932) on Monday to 12.57% (n = 2,709,129) on Thursday, showing less pronounced peaks than the ESKD group.

**Fig 3:**
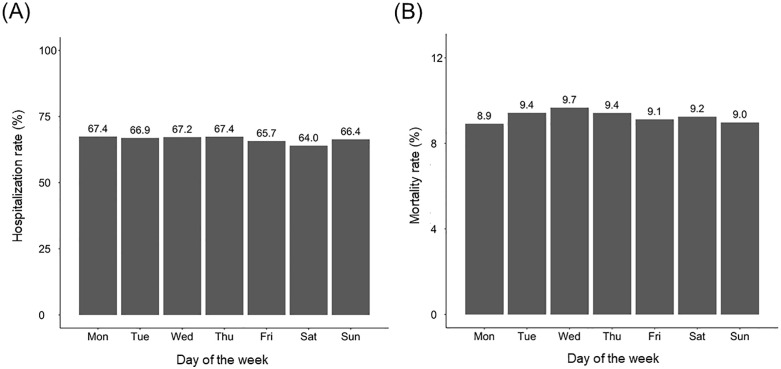
Hospitalization and mortality in end-stage kidney disease (ESKD) patients by emergency department (ED) visit day. (A) Hospitalization rates in patients with ESKD according to the day of ED visit. (B) In-hospital mortality rates in patients with ESKD according to the day of ED visit. Bars represent hospitalization/mortality rates in patients with ESKD by the day of emergency department visit.

Fig 3 provides a detailed analysis of hospitalization and in-hospital mortality rates for ED visits among patients with ESKD by the day of ED visit. For hospitalizations (Fig 3A), the highest rates were observed on Mondays, with 67.42% (n = 21,397) of ED visits leading to admission. Comparable rates were noted on other weekdays, including 67.38% (n = 14,489) on Thursdays and 67.21% (n = 15,005) on Wednesdays. The lowest hospitalization rate was on Saturdays (63.99%, n = 12,114), slightly higher on Sundays (66.37%, n = 11,610), but it remained lower than those on weekdays. Bonferroni post hoc analysis indicated significant differences between Saturdays and other weekdays, particularly when compared to Mondays (p < 0.001), Tuesdays (p < 0.001), Wednesdays (p < 0.001), Thursdays (p < 0.001), and Fridays (p = 0.005). Differences between Sundays and other weekdays were less pronounced, with only Saturdays showing a significant difference (p < 0.001).

For in-hospital mortality (Fig 3B), the highest rates occurred during Wednesday ED visits at 9.67% (n = 1,451), followed by Tuesdays at 9.43% (n = 1,589) and Thursdays at 9.42% (n = 1,365). Saturday and Friday ED visits showed slightly lower mortality rates at 9.25% (n = 1,120) and 9.11% (n = 1,336), respectively. The lowest mortality rates were observed on Mondays (8.92%, n = 1,908) and Sundays ED visits (8.98%, n = 1,042). Statistical analysis revealed no significant differences in in-hospital mortality across the week, indicating consistent outcomes irrespective of admission timing. These findings highlight distinct temporal patterns in hospitalization rates, with a significant reduction on Saturdays compared to other weekdays, as confirmed by Bonferroni post hoc analysis (data not shown) (Fig 3).

### Weekly distribution of emergency department visits, hospitalization rates, and in-hospital mortality for major conditions in end-stage kidney disease patients

Table 2 details information on ED visits, hospitalization rates, and in-hospital mortality for major conditions according to the ICD-10 code in patients with ESKD across different days of the week. For ED visits, the most common reasons included acute and chronic kidney failure (ICD-10 diagnosis code N17 or N18), accounting for 42.03% of ED visits on Mondays (n = 13,332), followed by other causes at 28.4% (n = 9,009). Conditions such as infection (7.54%, n = 2,392) and heart failure with pulmonary edema (7.23%, n = 2,294) were also significant contributors on Mondays. In contrast, Sundays were associated with decreased acute and chronic kidney failure-related visits at 37.64% (n = 6,573), with relatively higher proportions of other conditions (31.68%, n = 5,533). For hospitalization rates, patients with heart failure with pulmonary edema had the highest admission rates, which peaked on Wednesdays at 88.00% and declined slightly to 80.02% on Saturdays.

**Table 2:** Weekly distribution of ED visits, hospitalization rates, and in-hospital mortality for major conditions in patients with ESKD.

Cause of ED visits (%), Diagnosis code by ICD-10	Mon	Tue	Wed	Thu	Fri	Sat	Sun
Heart failure and pulmonary edema	7.23	5.99	5.16	5.18	4.92	5.32	9.34
Ischemic heart disease	2.09	2.15	2.15	2.2	2.11	1.99	2.16
Cerebrovascular disease	1.89	1.93	2.25	2.17	2.13	2.08	2.02
Infection	7.54	8.14	8.43	8.43	8.52	9.05	8.9
Electrolyte imbalance	2.82	2.27	1.55	1.68	1.61	1.62	3.47
Vascular access	4.21	4.09	4.47	4.5	4.99	4.03	0.94
GI problem	2.75	2.94	3.43	3.17	3.09	3.25	2.61
Fracture	1.03	1.12	1.17	1.18	1.06	1.1	1.24
Others	28.4	29.92	30.28	30.42	30.28	31.6	31.68
Acute and chronic kidney failure	42.03	41.45	41.1	41.07	41.3	39.95	37.64
**Hospitalization rates (%)**	**Mon**	**Tue**	**Wed**	**Thu**	**Fri**	**Sat**	**Sun**
Heart failure and pulmonary edema	81.78	82.74	88	85.7	85.11	83.61	80.02
Ischemic heart disease	90.96	90.19	89.38	89.43	86.14	88.03	86.74
Cerebrovascular disease	93.65	91.94	94.82	93.36	94.3	94.42	93.75
Infection	83.65	83.53	83.03	83.16	84.18	80.04	78.14
Electrolyte imbalance	81.58	81.05	83.19	85.32	83.24	83.06	74.26
Vascular access	48.43	44.75	45.59	44.72	47.84	63.56	79.27
GI problem	93.24	93.5	91.38	92.38	93.03	92.52	92.54
Fracture	95.12	97.51	96.18	97.23	99.58	96.65	95.85
Others	74.61	73.18	72.95	73.24	73.38	68.91	69.8
Acute and chronic kidney failure	53.31	53.06	53.21	53.71	49.99	47	50.84
**In-hospital mortality rate (%)**	**Mon**	**Tue**	**Wed**	**Thu**	**Fri**	**Sat**	**Sun**
Heart failure and pulmonary edema	5.92	8.11	8.4	9.02	8.48	10.1	7.27
Ischemic heart disease	11.09	9.03	10.96	10.17	10.15	10.27	7.95
Cerebrovascular disease	16.61	18.65	19.54	22.48	19.91	18.01	23.33
Infection	11.44	12.58	12.11	11.22	11.97	11.09	10.62
Electrolyte imbalance	4.38	9.31	8.01	5.19	8.05	8.63	4.22
Vascular access	2.16	2.17	1.98	3.24	1.69	1.44	2.31
GI problem	6.27	5.51	5.57	6.67	6.71	5.98	9.72
Fracture	10.9	9.12	10.32	10.16	8.47	8.42	9.13
Others	12.15	11.87	11.88	11.64	10.67	11.33	11.26
Acute and chronic kidney failure	6.53	6.86	7.36	6.59	6.77	6.7	5.86

Data are presented as percentages.

The ICD-10 is using the modified version of the International Classification of Diseases, 10th revision.

ED, emergency department; ESKD, end-stage kidney disease; GI, gastrointestinal.

Similarly, ischemic heart diseases demonstrated consistently high hospitalization rates, peaking on Mondays at 90.96% and being the lowest (86.14%) on Fridays. Conditions such as infection and electrolyte imbalances showed hospitalization rates approximately 80–85% throughout the week, with slightly lower rates on weekends. For in-hospital mortality, cardiovascular disease was associated with the highest mortality rates, particularly on Sunday (23.33%, n = 77) and Thursday ED visits (22.48%, n = 98). Ischemic heart disease had the highest mortality on Monday ED visits (11.09%, n = 67), while it was lower on Sunday ED visits (7.95%, n = 26). Mortality rates for infection were consistent, ranging from 11.09% among Saturday ED encounters to 12.58% on Tuesday. Electrolyte imbalance disorders showed fluctuations, with mortality rates as high as 9.31% among Tuesday ED visits and as low as 4.22% among Sunday ED visits.

Table 2 categorizes the primary diagnoses of ED visits based on discharge diagnosis codes using ICD-10. We included 159,291 patients, with a diagnosis code N185 (ESKD) present in either the discharge or hospital diagnosis in 159,456 patients. Among them, 165 patients were excluded owing to missing primary discharge diagnoses or unavailable discharge diagnosis codes, resulting in a discrepancy between the total number of patients with ESKD in Table 1 and in this analysis.

### Risk of hospitalization and in-hospital mortality according to the weekday in end-stage kidney disease patients

Table 3 summarizes hospitalization and in-hospital mortality risks in patients with ESKD based on ED visit days. Results, adjusted for various factors, are presented across three models: Model 1 (adjusted for age and sex), Model 2 (adjusted for age, sex, and KTAS score), and Model 3 (adjusted for age, sex, KTAS score, insurance status, and hospital level). Significant variations were observed for hospitalization depending on the day of the week. In Model 1, Saturdays showed the lowest likelihood of hospitalization compared to Sundays (OR 0.885, 95% CI 0.848–0.925, p < 0.001), and Fridays showed a reduced likelihood (OR 0.948, 95% CI 0.909–0.989, p = 0.014). When the KTAS score was added in Model 2, weekdays showed a significantly higher hospitalization risk, with Thursdays having the highest odds (OR 1.136, 95% CI 1.086–1.188, p < 0.001) followed by Wednesdays (OR 1.120, 95% CI 1.071–1.171, p < 0.001). This trend persisted in Model 3, with Thursdays (OR 1.146, 95% CI 1.095–1.199, p < 0.001) and Wednesdays (OR 1.128, 95% CI 1.078–1.179, p < 0.001) maintaining the highest hospitalization risks. Conversely, in-hospital mortality risk showed less pronounced differences across days. In Model 1, no significant variations in mortality risks were observed. However, in Model 2, Wednesdays (OR 1.133, 95% CI 1.040–1.234, p = 0.005) and Thursdays (OR 1.099, 95% CI 1.008–1.199, p = 0.033) displayed slightly higher mortality risks compared to Sundays. This trend persisted in Model 3, where Wednesdays (OR 1.135, 95% CI 1.042–1.237, p = 0.004) and Thursdays (OR 1.102, 95% CI 1.011–1.203, p = 0.028) remained associated with higher mortality risks.

**Table 3:** Risk of hospitalization and in-hospital mortality in patients with ESKD.

Model 1				
	Hospitalization		Mortality	
	OR (95% CI)	p-value	OR (95% CI)	p-value
Mon	1.022 (0.982–1.063)	0.285	0.955 (0.882–1.035)	0.258
Tue	0.995 (0.955–1.037)	0.804	1.007 (0.927–1.094)	0.873
Wed	1.019 (0.977–1.063)	0.388	1.048 (0.963–1.140)	0.279
Thu	1.022 (0.980–1.067)	0.313	1.016 (0.933–1.107)	0.713
Fri	0.948 (0.909–0.989)	0.014	0.977 (0.897–1.065)	0.598
Sat	0.885 (0.848–0.925)	<0.001	0.999 (0.914–1.092)	0.980
Sun	ref.		ref.	
**Model 2**				
	**Hospitalization**		**Mortality**	
	**OR (95% CI)**	**p-value**	**OR (95% CI)**	**p-value**
Mon	1.047 (1.004–1.092)	0.030	0.968 (0.893–1.051)	0.438
Tue	1.057 (1.012–1.104)	0.013	1.049 (0.964–1.141)	0.267
Wed	1.120 (1.071–1.171)	<0.001	1.133 (1.040–1.234)	0.005
Thu	1.136 (1.086–1.188)	<0.001	1.099 (1.008–1.199)	0.033
Fri	1.059 (1.013–1.108)	0.011	1.057 (0.968–1.153)	0.216
Sat	0.971 (0.927–1.017)	0.211	1.054 (0.962–1.154)	0.260
Sun	ref.		ref.	
**Model 3**				
	**Hospitalization**		**Mortality**	
	**OR (95% CI)**	**p-value**	**OR (95% CI)**	**p-value**
Mon	1.054 (1.011–1.099)	0.013	0.970 (0.894–1.052)	0.460
Tue	1.065 (1.020–1.113)	0.004	1.051 (0.966–1.144)	0.246
Wed	1.128 (1.078–1.179)	<0.001	1.135 (1.042–1.237)	0.004
Thu	1.146 (1.095–1.199)	<0.001	1.102 (1.011–1.203)	0.028
Fri	1.070 (1.023–1.118)	0.003	1.060 (0.972–1.157)	0.189
Sat	0.977 (0.933–1.023)	0.318	1.055 (0.963–1.155)	0.249
Sun	ref.		ref.	

Model 1, Adjusted age, sex; Model 2, Model 1 + Adjusted KTAS score; Model 3, Model 2 + Adjusted Health-care Insurance, Hospital level.

CI, confidence interval; ESKD, end-stage kidney disease; KTAS, Korean Triage and Acuity Scale; OR, odds ratio.

## Discussion

Herein, we found that patients with ESKD presented to the ED on Mondays and Tuesdays more frequently than those without CKD. The hospitalization rate was the highest among Monday ED visits, while the in-hospital mortality rate was the highest among Wednesday ED visits. The most common reason for ED visits was kidney disease-specific conditions, but hospitalization rates were higher for other disease conditions. After controlling for several potential confounders, mid-weekday ED visits (Wednesdays and Thursdays) had the highest adjusted risks for hospitalization and in-hospital mortality.

Previous studies reported that patients with ESKD visited the ED more frequently on Mondays or Tuesdays, that is, after weekends or extended interdialytic periods [3,8–10]. Consistently, herein, patients with ESKD visited the ED more frequently on Mondays and Tuesdays than those without CKD. Among the ED visits involving patients with ESKD, those on Mondays and Tuesdays had the highest percentage. Patients visiting the ED on Mondays and Tuesdays were mostly older men (≥60 years), had medical insurance, and a more severe KTAS classification (resuscitation or emergent) compared to those visiting the ED on other days. The highest ED-visit rate on Mondays and Tuesdays is known as the “post-weekend effect,” which is thought to be related to the in-center hemodialysis schedule [5,15–17]. In the Republic of Korea, patients on hemodialysis account for 75% of prevalent ESKD cases, while those on peritoneal dialysis account for only 5.5% [18]. Therefore, most ED visits in NEDIS involve patients on hemodialysis. The typical in-center hemodialysis schedule is thrice-weekly: either Monday-Wednesday-Friday or Tuesday-Thursday-Saturday. The interdialytic interval is usually 48 h, but on weekends, it extends to 72 h (from Friday to Monday or Saturday to Tuesday). A longer interdialytic interval may lead to fluid accumulation and electrolyte imbalances. These physiologic changes increase the likelihood of requiring emergent care on Mondays or Tuesdays. Corroborating our findings, a study conducted in 15 European countries revealed that missed dialysis sessions, especially after the extended interdialytic interval, were significantly associated with increased hospitalization and mortality rates [19]. The mortality rate surged after a missed first session following a long gap (48–72 h), emphasizing the critical need for timely dialysis to avoid these adverse outcomes. The surge in mortality after missed dialysis sessions aligns with the post-weekend increase in healthcare utilization observed in our study, highlighting the importance of addressing non-adherence, particularly in patients who miss dialysis sessions owing to logistical or social factors. These findings highlight that the “post-weekend effect” observed in hemodialysis patients may be attributed to the prolonged interdialytic interval, fluid overload, and cardiovascular instability following the longer interdialytic interval over the weekend. Furthermore, reduced healthcare accessibility during weekends may exacerbate clinical deterioration, leading to increased emergency visits early in the week. The midweek peak in hospitalization and mortality likely reflects the cumulative burden of delayed care and clustering of high-acuity cases after weekends.

Regarding reasons for ED visits, kidney disease-specific conditions were more prevalent in patients visiting on Mondays and Tuesdays than in those on other days. The exact disease conditions related to kidney disease could not be assessed in the database since only ICD-10 codes were extracted. We presume that kidney disease-specific conditions included volume overload, electrolyte imbalances (e.g., hyperkaliemia), hypertension, or acidosis, which were manageable in the ED. Patients with more severe conditions associated with heart failure, electrolyte imbalances, or acid-base disturbances would have been given a diagnostic code for such conditions. Although hospitalization rate was the highest on Mondays, neither Mondays nor Tuesdays exhibited the highest adjusted risk for hospitalization. This suggests that the reasons for ED visits on such days were potentially preventable. Potentially preventable kidney disease-related ambulatory care-sensitive conditions include hyperkaliemia, heart failure, volume overload, and malignant hypertension [20]. Acknowledging the high post-weekend ED utilization rate among patients with ESKD and identifying the risk factors for potentially preventable ED utilization would help allocate healthcare resources efficiently and improve preventive strategies for acute care in patients with ESKD [21].

Our findings revealed lower hospitalization rates among patients with ESKD visiting the ED on weekends than among those visiting the ED on weekdays, consistent with previous reports [5,22]. The lower hospitalization rate on weekends than on weekdays may reflect higher weekend ED visits by patients with non-urgent symptoms due to clinic closures. Regarding major conditions for ED visits, the percentage of “other conditions” was higher on weekends than on weekdays, including non-specific chest pain, dizziness, and hypoglycemia. In addition, the reason for ED visit might have been related to infectious status, especially coronavirus disease (COVID-19). The study period included the COVID-19 outbreak; therefore, when we compared ED visits from 2018–2019 and 2020–2021 by pandemic year, we found that hemodialysis patients visited ED more often in 2020–2021 during the COVID-19 outbreak than in 2018–2019 (76,277 visits in 2018–2019 vs. 83,179 visits in 2020–2021). While ED visits in the general population have tended to decrease during COVID-19 [23], this vulnerable population may visit the ED to confirm their infectious status or other related conditions. Further comparison of ED visits during COVID-19 is needed to clarify the difference between ESKD patients and the general population.

However, the hospitalization and mortality rates and risks differed from those in previous studies. First, in this study, the weekday hospitalization rate differed from those presented in previous reports [5,22], which showed that hospitalization rate was the highest among ED visits on Mondays and Tuesdays. Herein, the crude hospitalization rate and age- and sex-adjusted risk for hospitalization were highest on Mondays and Thursdays. However, after adjusting for the KTAS score, insurance, and hospital level, the risk of hospitalization was highest on Thursdays and Wednesdays. Second, the in-hospital mortality rate also differed from previous reports. For instance, Sakhuja et al. [4] analyzed claim data from the United States and found that patients with ESKD admitted on weekends had higher mortality rates than those admitted on weekdays. Fotheringham et al. [22] analyzed data from the UK Renal Registry and found that mortality rates were higher on Mondays and Tuesdays than on other days. In our study, the crude in-hospital mortality rate was highest in ED encounters on Wednesdays, followed by those on Tuesdays and Thursdays.

Adjusted risk for mortality was the highest on Wednesdays and Thursdays in all three models. This inconsistency may reflect different healthcare accessibility systems and patient care-seeking behavior. The Republic of Korea has better healthcare accessibility than other countries because of its geographical characteristics and national medical insurance system. Therefore, many patients can receive healthcare without seeking the ED, even on Saturdays, with a relatively low financial burden for medical costs. We speculate that patients with ESKD who visited the ED in the mid-week had severe disease that could not be managed at outpatient clinics and needed direct hospitalization via the ED. The higher severity of mid-week ED cases can be explained by the patterns of the arrival route to the ED and the percentage of major conditions related to the ED visit. Patients transferred from other hospitals or outpatient clinics were more prevalent among ED encounters on Wednesdays and Thursdays than on other days. Patients who visited the ED on Wednesdays and Thursdays and were hospitalized or died had relatively higher percentages of heart failure with pulmonary edema, ischemic heart disease, and cerebrovascular disease than those visiting the ED on other days. The weekly ED visit pattern was similar to those in other countries, while the hospitalization and mortality patterns differed. These findings suggest that the disposition and outcomes of ED visits are not solely the consequence of illness but also reflect the healthcare-seeking behavior of patients, access to healthcare, and medical insurance [24,25]. Although several studies have investigated ED utilization among dialysis patients in Asia, most have been limited to single-center settings or lacked detailed analysis of temporal patterns across the week. For instance, a population-based study in Taiwan reported high ED utilization among patients receiving maintenance dialysis, but did not explore weekly or weekday–weekend variation [26]. To the best of our knowledge, no large-scale nationwide study from Asia has comprehensively examined weekly trends in ED visits, hospitalizations, and in-hospital mortality among patients with ESKD. Our study therefore fills a critical gap in the literature and provides region-specific insights that may guide healthcare planning and policy.

This study has some limitations. First, the data did not include the exact ED visit and dialysis session time. Information on whether patients on hemodialysis visited the ED before or after the dialysis session was not available in the NEDIS database. Second, since we used ICD-10 diagnostic codes to assess the major conditions related to ED visits, an accurate diagnosis at the time of each ED visit might not have been possible. For example, why “acute kidney failure” was the cause of ED visits in patients with ESKD remains unknown. The reliance on ICD-10 codes for diagnosis, such as N18.5, can lead to misclassification owing to potential biases in coding practices and inherent limitations of using a standardized coding system for complex medical situations. These limitations could impact the accuracy of research findings and other clinical outcomes. Third, the administrative data used in this study were observational rather than randomized, causing limitations to inferring causality. The retrospective study design might have influenced bias in data recording and patient selection, and other confounding variables might have influenced the results, such as demographic and laboratory parameters not included in the NEDIS database. Fourth, each case in the NEDIS database referred to an ED visit, but not a patient who visited the ED; therefore, we could not adjust for repeat visits by the same individual. Finally, in this NEDIS dataset, we were unable to distinguish between patients receiving hemodialysis and those receiving peritoneal dialysis. However, in South Korea, more than 85% of patients with ESKD are on hemodialysis, and the percentage of those on peritoneal dialysis has been decreasing in recent years [27]. In addition, data on comorbidities were not available in the NEDIS database. However, this study is the first to explore the weekly ED utilization pattern in patients with ESKD in the Republic of Korea. Moreover, we analyzed a large number ED visits from a nationwide cohort and adjusted the results for variables such as visit severity and insurance level.

## Conclusions

In conclusion, after the longer interdialytic interval over the weekend, patients with ESKD visited the ED more frequently on Mondays and Tuesdays. Hospitalization and in-hospital mortality risks were the highest in midweek (Wednesdays and Thursdays), reflecting the impact of illness severity and access to healthcare in the Republic of Korea. Thus, incorporating ED utilization patterns into healthcare resource planning and policy development is crucial for improving acute care for patients with ESKD and reducing the burden on ED services.

## Supporting information

S1 Table ICD-10 Codes for causes of emergency department visit. (DOCX)

## References

[1] Han G, Bohmart A, Shaaban H, Mages K, Jedlicka C, Zhang Y, et al. Emergency department utilization among maintenance hemodialysis patients: a systematic review. Kidney Med. 2021;4(2):100391. doi: 10.1016/j.xkme.2021.09.007 35243303 PMC8861946

[2] Wang N, Pei J, Fan H, Ali Y, Prushinskaya A, Zhao J, et al. Emergency department use by patients with end-stage renal disease in the United States. BMC Emerg Med. 2021;21(1):25. doi: 10.1186/s12873-021-00420-8 33653282 PMC7927369

[3] Harel Z, Wald R, McArthur E, Chertow GM, Harel S, Gruneir A, et al. Rehospitalizations and emergency department visits after hospital discharge in patients receiving maintenance hemodialysis. J Am Soc Nephrol. 2015;26(12):3141–50. doi: 10.1681/ASN.2014060614 25855772 PMC4657827

[4] Sakhuja A, Schold JD, Kumar G, Dall A, Sood P, Navaneethan SD. Outcomes of patients receiving maintenance dialysis admitted over weekends. Am J Kidney Dis. 2013;62(4):763–70. doi: 10.1053/j.ajkd.2013.03.014 23669002 PMC3783620

[5] Zhang S, Morgenstern H, Albertus P, Nallamothu BK, He K, Saran R. Emergency department visits and hospitalizations among hemodialysis patients by day of the week and dialysis schedule in the United States. PLoS One. 2019;14(8):e0220966. doi: 10.1371/journal.pone.0220966 31415609 PMC6695146

[6] Syed ST, Gerber BS, Sharp LK. Traveling towards disease: transportation barriers to health care access. J Commun Health. 2013;38(5):976–93. doi: 10.1007/s10900-013-9681-1 23543372 PMC4265215

[7] Foley RN, Gilbertson DT, Murray T, Collins AJ. Long interdialytic interval and mortality among patients receiving hemodialysis. N Engl J Med. 2011;365(12):1099–107. doi: 10.1056/NEJMoa1103313 21992122

[8] Golestaneh L, Karaboyas A, Cavanaugh K, Umeukeje EM, Johns TS, Thorpe RJ Jr, et al. The role of place in disparities affecting black men receiving hemodialysis. Kidney Int Rep. 2020;6(2):357–65. doi: 10.1016/j.ekir.2020.10.014 33615061 PMC7879205

[9] Yoo KD, Chao C-T, Lee JP, Abu-Alfa AK. The role of international renal disaster preparedness working groups in difficult settings: bridge over troubled water. Curr Opin Nephrol Hypertens. 2024;33(6):636–40. doi: 10.1097/MNH.0000000000001024 39234876

[10] ChoAJ, JeongSA, ParkHC, YoonHE, KimJ, LeeY-K, et al. Emergency department visits for patients with end-stage kidney disease in Korea: registry data from the National Emergency Department Information System 2019-2021. Kidney Res Clin Pract. 2025. doi: 10.23876/j.krcp.24.170 39849858

[11] Yoo HH, Ro YS, Ko E, Lee J-H, Han S-H, Kim T, et al. Epidemiologic trends of patients who visited nationwide emergency departments: a report from the National Emergency Department Information System (NEDIS) of Korea, 2018-2022. Clin Exp Emerg Med. 2023;10(S):S1–12. doi: 10.15441/ceem.23.151 37967858 PMC10662522

[12] Cho Aj, Yoo KD, Yoon HE, Jeong SA, Choi W, Choi DH, et al. Impact of health insurance status on hospitalization and mortality from emergency department admission in patients with end-stage kidney disease: a Korean nationwide registry analysis. Kidney Res Clin Pract. 2025;:10.23876/j.krcp.25.184. doi: 10.23876/j.krcp.25.184 40994421 PMC13176912

[13] National Emergency Medical Center (NEMC SKI. [cited 2024 Aug 14] Establishing and operating an emergency medical monitoring system. Available from: https://wwwe-genorkr/nemc/business_othersdo?con-tents-no=77

[14] Kwon H, Kim YJ, Jo YH, Lee JH, Lee JH, Kim J, et al. The Korean Triage and Acuity Scale: associations with admission, disposition, mortality and length of stay in the emergency department. Int J Qual Health Care. 2019;31(6):449–55. doi: 10.1093/intqhc/mzy184 30165654

[15] Komenda P, Tangri N, Klajncar E, Eng A, Di Nella M, Hiebert B, et al. Patterns of emergency department utilization by patients on chronic dialysis: a population-based study. PLoS One. 2018;13(4):e0195323. doi: 10.1371/journal.pone.0195323 29664922 PMC5903639

[16] Chien C-W, Huang C-J, Chao Z-H, Huang S-K, Chen P-E, Tung T-H. Hemodialysis interval and its association with emergency care and mortality: a nationwide population-based cohort study. Medicine (Baltimore). 2019;98(10):e14816. doi: 10.1097/MD.0000000000014816 30855505 PMC6417509

[17] Uwumiro FE, Okpujie VO, Oyesomi A, Madu FC, Ilelaboye A, Shielu ML, et al. Weekend effect on mortality, access to renal replacement therapy, and other outcomes among patients with end-stage renal disease: a retrospective analysis of the nationwide inpatient sample. Cureus. 2023;15(1):e34139. doi: 10.7759/cureus.34139 36843711 PMC9948686

[18] Hong YA, Ban TH, Kang C-Y, Hwang SD, Choi SR, Lee H, et al. Trends in epidemiologic characteristics of end-stage renal disease from 2019 Korean Renal Data System (KORDS). Kidney Res Clin Pract. 2021;40(1):52–61. doi: 10.23876/j.krcp.20.202 33789383 PMC8041639

[19] Fotheringham J, Smith MT, Froissart M, Kronenberg F, Stenvinkel P, Floege J, et al. Hospitalization and mortality following non-attendance for hemodialysis according to dialysis day of the week: a European cohort study. BMC Nephrol. 2020;21(1):218. doi: 10.1186/s12882-020-01874-x 32517695 PMC7285433

[20] Ronksley PE, Scory TD, McRae AD, MacRae JM, Manns BJ, Lang E, et al. Emergency department use among adults receiving dialysis. JAMA Netw Open. 2024;7(5):e2413754. doi: 10.1001/jamanetworkopen.2024.13754 38809552 PMC11137633

[21] Tennankore KK, Nadeau-Fredette A-C, Matheson K, Chan CT, Trinh E, Perl J. Home versus in-center dialysis and day of the week hospitalization: a cohort study. Kidney360. 2021;3(1):103–12. doi: 10.34067/KID.0003552021 35368556 PMC8967598

[22] Fotheringham J, Fogarty DG, El Nahas M, Campbell MJ, Farrington K. The mortality and hospitalization rates associated with the long interdialytic gap in thrice-weekly hemodialysis patients. Kidney Int. 2015;88(3):569–75. doi: 10.1038/ki.2015.141 25970155

[23] Kim SJ, Kim H, Park YH, Kang CY, Ro YS, Kim OH. Analysis of the impact of the coronavirus disease epidemic on the emergency medical system in South Korea using the Korean Triage and Acuity Scale. Yonsei Med J. 2021;62(7):631–9. doi: 10.3349/ymj.2021.62.7.631 34164961 PMC8236346

[24] Varney J, Weiland TJ, Jelinek G. Efficacy of hospital in the home services providing care for patients admitted from emergency departments: an integrative review. Int J Evid Based Healthc. 2014;12(2):128–41. doi: 10.1097/XEB.0000000000000011 24945961

[25] Jang Y, Kim SG, Lee S, Oh HH, Shin N, Lee Y-K, et al. Disaster emergency meal plans for Korean patients who require hemodialysis. Kidney Res Clin Pract. 2025;44(2):228–37. doi: 10.23876/j.krcp.24.242 39849857 PMC11985318

[26] Lin Y-C, Hsu H-K, Lai T-S, Chiang W-C, Lin S-L, Chen Y-M, et al. Emergency department utilization and resuscitation rate among patients receiving maintenance hemodialysis. J Formos Med Assoc. 2019;118(12):1652–60. doi: 10.1016/j.jfma.2019.01.007 30711255

[27] Kim KM, Jeong SA, Ban TH, Hong YA, Hwang SD, Choi SR, et al. Status and trends in epidemiologic characteristics of diabetic end-stage renal disease: an analysis of the 2021 Korean Renal Data System. Kidney Res Clin Pract. 2024;43(1):20–32. doi: 10.23876/j.krcp.23.130 38268124 PMC10846995

