# Supplementary material for: Weekly trends in emergency department visits, hospitalizations, and mortality in patients with end-stage kidney disease: nationwide registry analysis
Source: PLoS One. 2026 Feb 20;21(2):e0339905. doi: 10.1371/journal.pone.0339905 (PMC12922999; doi:10.1371/journal.pone.0339905)
Supplement: S1 Table — (DOCX) [file pone.0339905.s001.docx]

**S1 Table. ICD-10 Codes for causes of emergency department visit.**

| Heart failure and pulmonary edema | I50, J81, R06 |
| --- | --- |
| Ischemic heart disease | I20-25 |
| Cerebrovascular disease | I60-69 |
| Infection | A00-A09, A30-A49, B95-B97, B99, A15-A19, G00-G09, J00-06, J09-J18, J20-J22, J30-J39, J85-J86, K61, K81, N10, N30, N39.0,  R50 |
| Electrolyte imbalance | E87 |
| Vascular access | T82 |
| GI problem | K92, A09 |
| Fracture | S72 |
